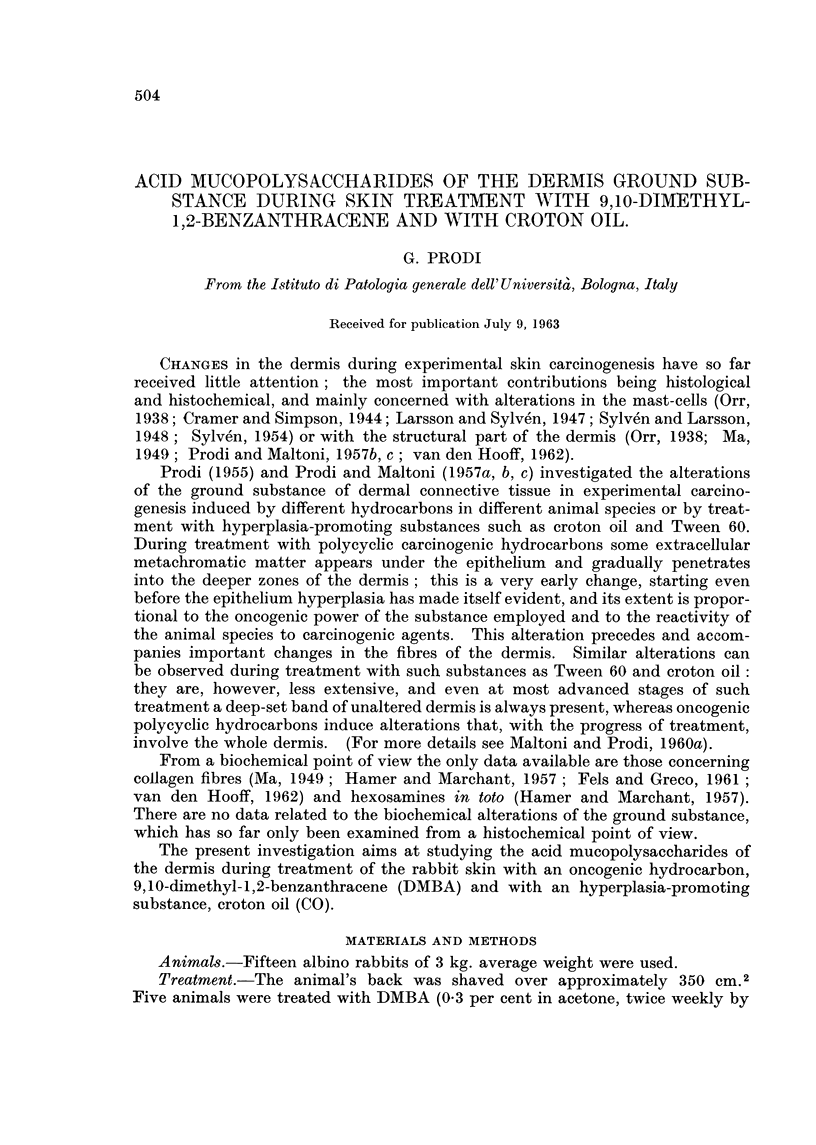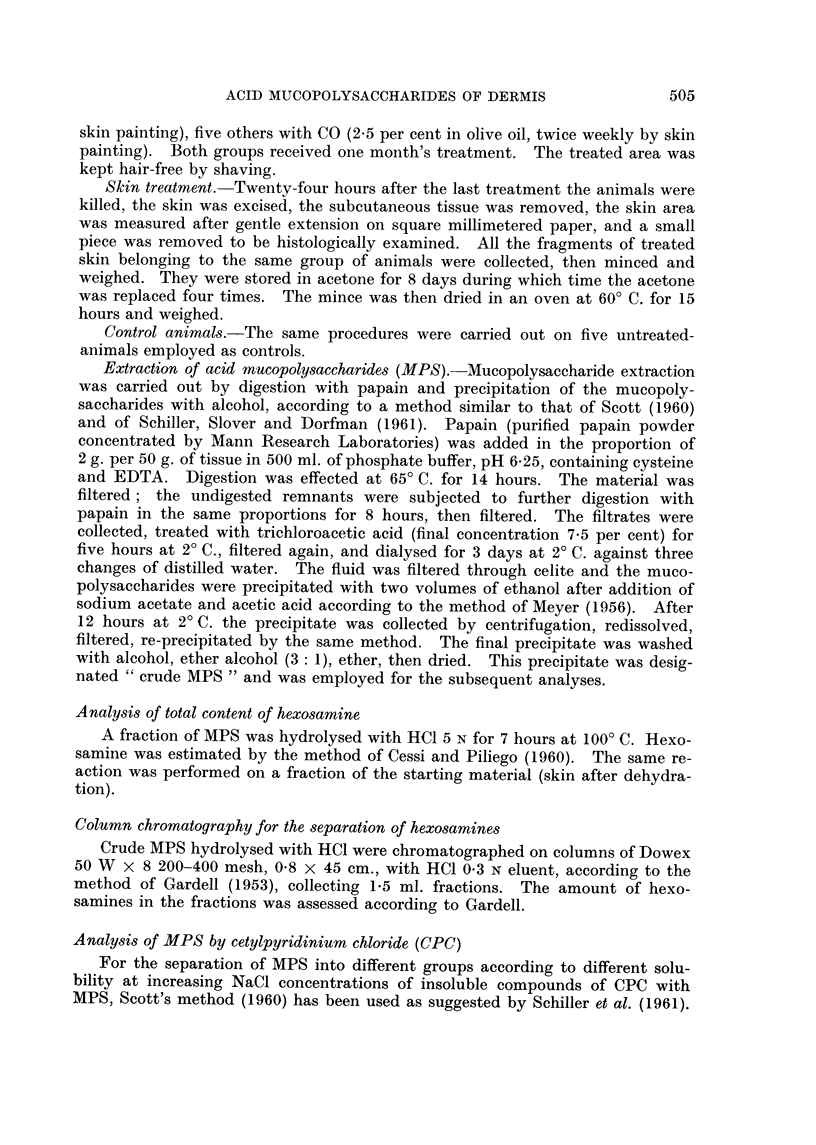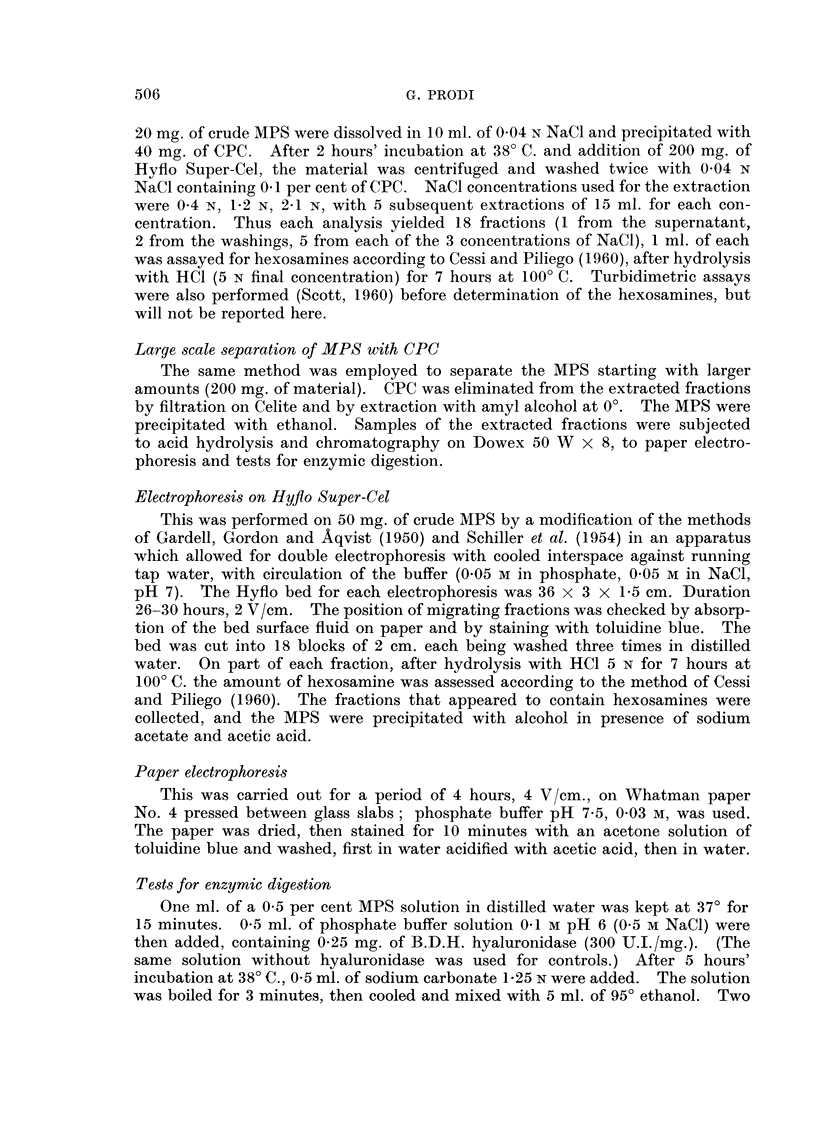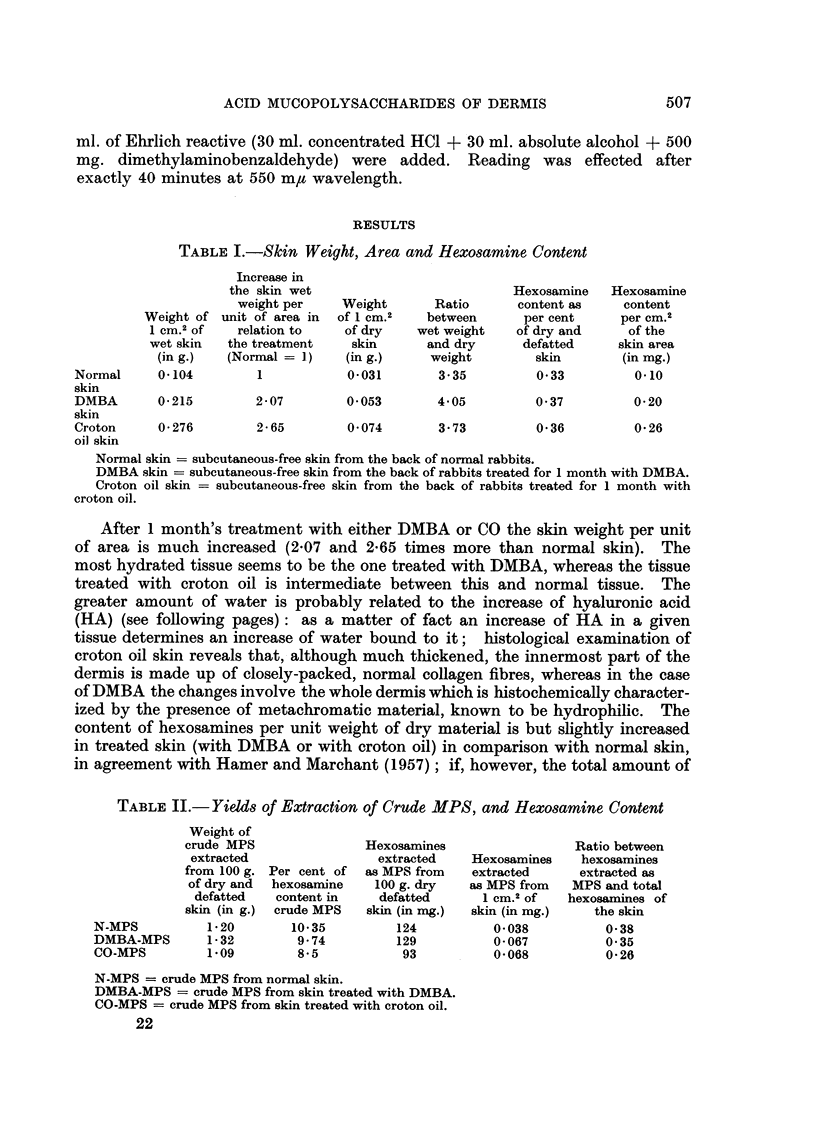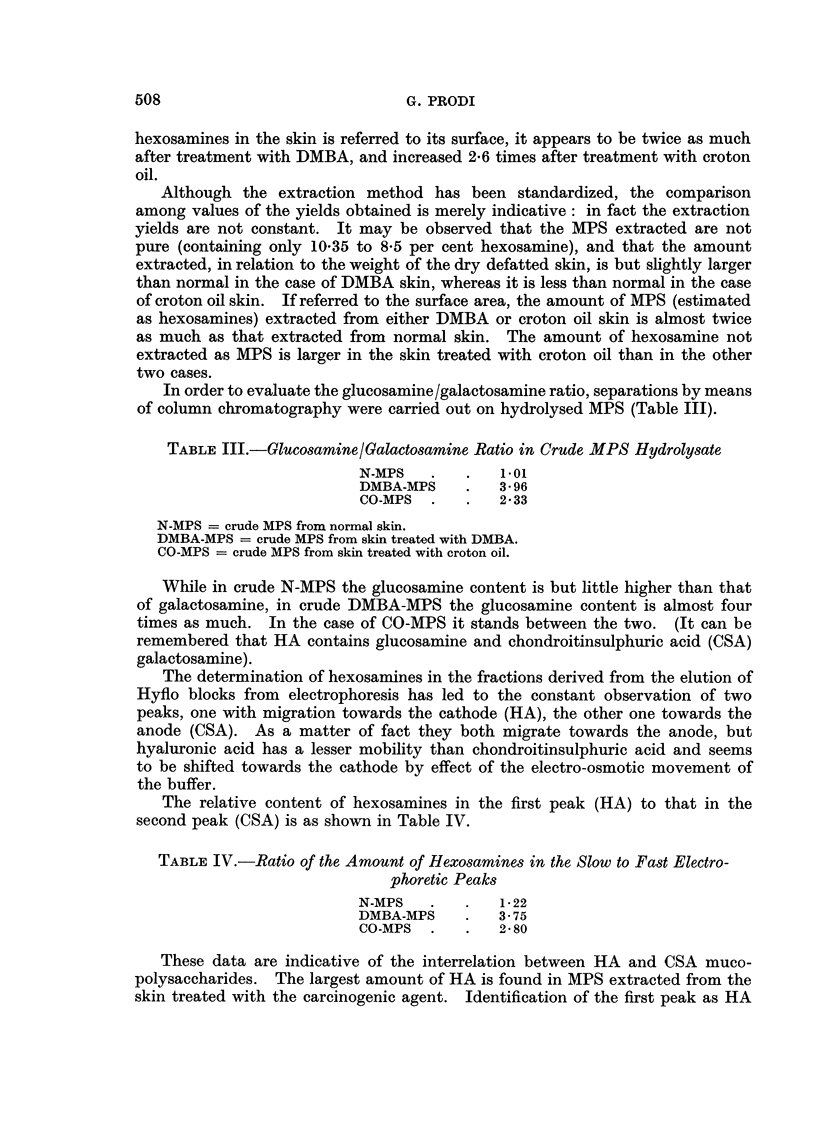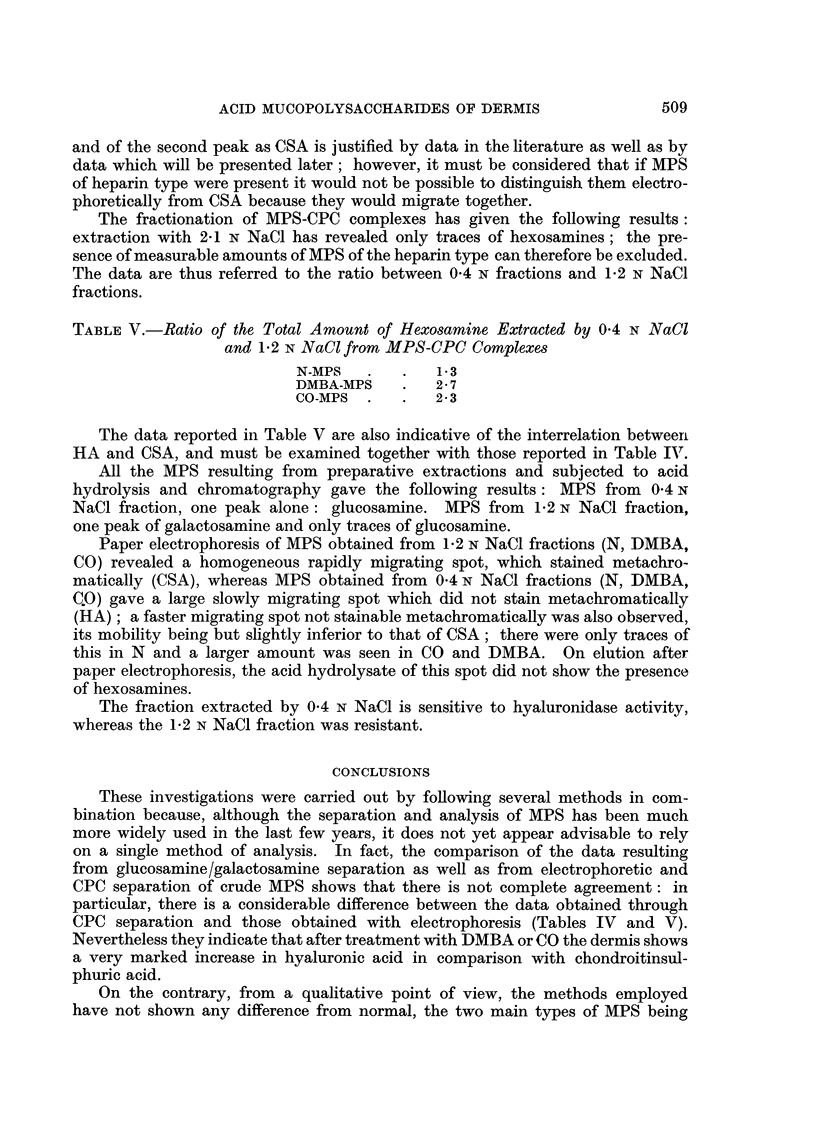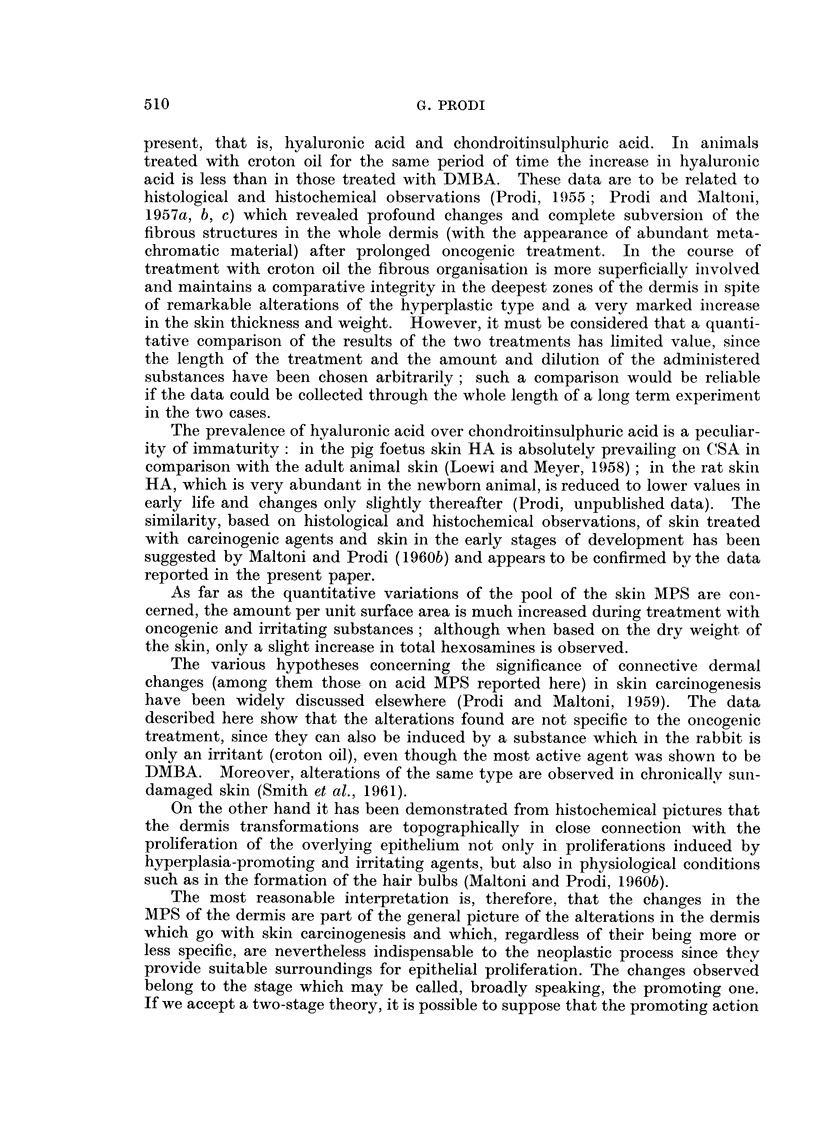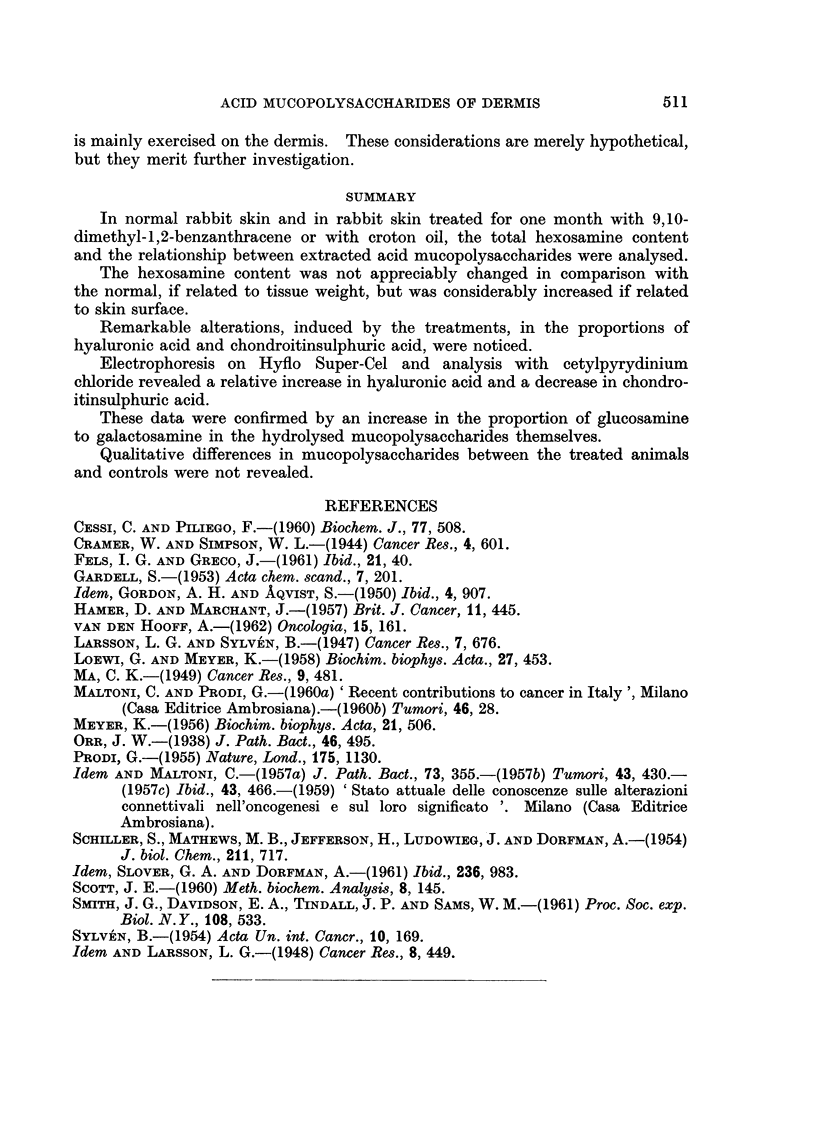# Acid Mucopolysaccharides of the Dermis Ground Substance During Skin Treatment with 9,10-Dimethyl-1,2-Benzanthracene and with Croton Oil

**DOI:** 10.1038/bjc.1963.68

**Published:** 1963-09

**Authors:** G. Prodi


### ACID MUCOPOLYSACCHARIDES OF THE DERMIS GROUND SUBSTANCE DURING SKIN TREATMENT WITH 9,10-DIMETHYL-1,2-BENZANTHRACENE AND WITH CROTON OIL.

G. PRODI

*From the Istituto di Patologia generale dell'Università, Bologna, Italy*

Received for publication July 9, 1963

CHANGES in the dermis during experimental skin carcinogenesis have so far received little attention ; the most important contributions being histological and histochemical, and mainly concerned with alterations in the mast-cells (Orr, 1938 ; Cramer and Simpson, 1944 ; Larsson and Sylvén, 1947 ; Sylvén and Larsson, 1948 ; Sylvén, 1954) or with the structural part of the dermis (Orr, 1938; Ma, 1949 ; Prodi and Maltoni, 1957*b*, *c* ; van den Hooff, 1962).

Prodi (1955) and Prodi and Maltoni (1957*a*, *b*, *c*) investigated the alterations of the ground substance of dermal connective tissue in experimental carcinogenesis induced by different hydrocarbons in different animal species or by treatment with hyperplasia-promoting substances such as croton oil and Tween 60. During treatment with polycyclic carcinogenic hydrocarbons some extracellular metachromatic matter appears under the epithelium and gradually penetrates into the deeper zones of the dermis ; this is a very early change, starting even before the epithelium hyperplasia has made itself evident, and its extent is proportional to the oncogenic power of the substance employed and to the reactivity of the animal species to carcinogenic agents. This alteration precedes and accompanies important changes in the fibres of the dermis. Similar alterations can be observed during treatment with such substances as Tween 60 and croton oil : they are, however, less extensive, and even at most advanced stages of such treatment a deep-set band of unaltered dermis is always present, whereas oncogenic polycyclic hydrocarbons induce alterations that, with the progress of treatment, involve the whole dermis. (For more details see Maltoni and Prodi, 1960*a*).

From a biochemical point of view the only data available are those concerning collagen fibres (Ma, 1949 ; Hamer and Marchant, 1957 ; Fels and Greco, 1961 ; van den Hooff, 1962) and hexosamines *in toto* (Hamer and Marchant, 1957). There are no data related to the biochemical alterations of the ground substance, which has so far only been examined from a histochemical point of view.

The present investigation aims at studying the acid mucopolysaccharides of the dermis during treatment of the rabbit skin with an oncogenic hydrocarbon, 9,10-dimethyl-1,2-benzanthracene (DMBA) and with an hyperplasia-promoting substance, croton oil (CO).

### MATERIALS AND METHODS

*Animals.*—Fifteen albino rabbits of 3 kg. average weight were used.

*Treatment.*—The animal's back was shaved over approximately 350 $cm.^2$ Five animals were treated with DMBA (0·3 per cent in acetone, twice weekly by skin painting), five others with CO (2·5 per cent in olive oil, twice weekly by skin painting). Both groups received one month's treatment. The treated area was kept hair-free by shaving.

*Skin treatment.*—Twenty-four hours after the last treatment the animals were killed, the skin was excised, the subcutaneous tissue was removed, the skin area was measured after gentle extension on square millimetered paper, and a small piece was removed to be histologically examined. All the fragments of treated skin belonging to the same group of animals were collected, then minced and weighed. They were stored in acetone for 8 days during which time the acetone was replaced four times. The mince was then dried in an oven at 60° C. for 15 hours and weighed.

*Control animals.*—The same procedures were carried out on five untreated-animals employed as controls.

*Extraction of acid mucopolysaccharides (MPS).*—Mucopolysaccharide extraction was carried out by digestion with papain and precipitation of the mucopolysaccharides with alcohol, according to a method similar to that of Scott (1960) and of Schiller, Slover and Dorfman (1961). Papain (purified papain powder concentrated by Mann Research Laboratories) was added in the proportion of 2 g. per 50 g. of tissue in 500 ml. of phosphate buffer, pH 6·25, containing cysteine and EDTA. Digestion was effected at 65° C. for 14 hours. The material was filtered; the undigested remnants were subjected to further digestion with papain in the same proportions for 8 hours, then filtered. The filtrates were collected, treated with trichloroacetic acid (final concentration 7·5 per cent) for five hours at 2° C., filtered again, and dialysed for 3 days at 2° C. against three changes of distilled water. The fluid was filtered through celite and the mucopolysaccharides were precipitated with two volumes of ethanol after addition of sodium acetate and acetic acid according to the method of Meyer (1956). After 12 hours at 2° C. the precipitate was collected by centrifugation, redissolved, filtered, re-precipitated by the same method. The final precipitate was washed with alcohol, ether alcohol (3 : 1), ether, then dried. This precipitate was designated " crude MPS " and was employed for the subsequent analyses.

### *Analysis of total content of hexosamine*

A fraction of MPS was hydrolysed with HCl 5 N for 7 hours at 100° C. Hexosamine was estimated by the method of Cessi and Piliego (1960). The same reaction was performed on a fraction of the starting material (skin after dehydration).

### *Column chromatography for the separation of hexosamines*

Crude MPS hydrolysed with HCl were chromatographed on columns of Dowex 50 W × 8 200–400 mesh, 0·8 × 45 cm., with HCl 0·3 N eluent, according to the method of Gardell (1953), collecting 1·5 ml. fractions. The amount of hexosamines in the fractions was assessed according to Gardell.

### *Analysis of MPS by cetylpyridinium chloride (CPC)*

For the separation of MPS into different groups according to different solubility at increasing NaCl concentrations of insoluble compounds of CPC with MPS, Scott's method (1960) has been used as suggested by Schiller *et al.* (1961).

20 mg. of crude MPS were dissolved in 10 ml. of 0·04 N NaCl and precipitated with 40 mg. of CPC. After 2 hours' incubation at 38° C. and addition of 200 mg. of Hyflo Super-Cel, the material was centrifuged and washed twice with 0·04 N NaCl containing 0·1 per cent of CPC. NaCl concentrations used for the extraction were 0·4 N, 1·2 N, 2·1 N, with 5 subsequent extractions of 15 ml. for each concentration. Thus each analysis yielded 18 fractions (1 from the supernatant, 2 from the washings, 5 from each of the 3 concentrations of NaCl), 1 ml. of each was assayed for hexosamines according to Cessi and Piliego (1960), after hydrolysis with HCl (5 N final concentration) for 7 hours at 100° C. Turbidimetric assays were also performed (Scott, 1960) before determination of the hexosamines, but will not be reported here.

*Large scale separation of MPS with CPC*

The same method was employed to separate the MPS starting with larger amounts (200 mg. of material). CPC was eliminated from the extracted fractions by filtration on Celite and by extraction with amyl alcohol at 0°. The MPS were precipitated with ethanol. Samples of the extracted fractions were subjected to acid hydrolysis and chromatography on Dowex 50 W × 8, to paper electrophoresis and tests for enzymic digestion.

*Electrophoresis on Hyflo Super-Cel*

This was performed on 50 mg. of crude MPS by a modification of the methods of Gardell, Gordon and Åqvist (1950) and Schiller *et al.* (1954) in an apparatus which allowed for double electrophoresis with cooled interspace against running tap water, with circulation of the buffer (0·05 M in phosphate, 0·05 M in NaCl, pH 7). The Hyflo bed for each electrophoresis was 36 × 3 × 1·5 cm. Duration 26–30 hours, 2 V/cm. The position of migrating fractions was checked by absorption of the bed surface fluid on paper and by staining with toluidine blue. The bed was cut into 18 blocks of 2 cm. each being washed three times in distilled water. On part of each fraction, after hydrolysis with HCl 5 N for 7 hours at 100° C. the amount of hexosamine was assessed according to the method of Cessi and Piliego (1960). The fractions that appeared to contain hexosamines were collected, and the MPS were precipitated with alcohol in presence of sodium acetate and acetic acid.

*Paper electrophoresis*

This was carried out for a period of 4 hours, 4 V/cm., on Whatman paper No. 4 pressed between glass slabs ; phosphate buffer pH 7·5, 0·03 M, was used. The paper was dried, then stained for 10 minutes with an acetone solution of toluidine blue and washed, first in water acidified with acetic acid, then in water.

*Tests for enzymic digestion*

One ml. of a 0·5 per cent MPS solution in distilled water was kept at 37° for 15 minutes. 0·5 ml. of phosphate buffer solution 0·1 M pH 6 (0·5 M NaCl) were then added, containing 0·25 mg. of B.D.H. hyaluronidase (300 U.I./mg.). (The same solution without hyaluronidase was used for controls.) After 5 hours' incubation at 38° C., 0·5 ml. of sodium carbonate 1·25 N were added. The solution was boiled for 3 minutes, then cooled and mixed with 5 ml. of 95° ethanol. Two ml. of Ehrlich reactive (30 ml. concentrated HCl + 30 ml. absolute alcohol + 500 mg. dimethylaminobenzaldehyde) were added. Reading was effected after exactly 40 minutes at 550 m$\mu$ wavelength.

### RESULTS

TABLE I.—*Skin Weight, Area and Hexosamine Content*

| | Weight of 1 cm.$^2$ of wet skin (in g.) | Increase in the skin wet weight per unit of area in relation to the treatment (Normal = 1) | Weight of 1 cm.$^2$ of dry skin (in g.) | Ratio between wet weight and dry weight | Hexosamine content as per cent of dry and defatted skin | Hexosamine content per cm.$^2$ of the skin area (in mg.) |
|---|---|---|---|---|---|---|
| Normal skin | 0·104 | 1 | 0·031 | 3·35 | 0·33 | 0·10 |
| DMBA skin | 0·215 | 2·07 | 0·053 | 4·05 | 0·37 | 0·20 |
| Croton oil skin | 0·276 | 2·65 | 0·074 | 3·73 | 0·36 | 0·26 |

Normal skin = subcutaneous-free skin from the back of normal rabbits.
DMBA skin = subcutaneous-free skin from the back of rabbits treated for 1 month with DMBA.
Croton oil skin = subcutaneous-free skin from the back of rabbits treated for 1 month with croton oil.

After 1 month's treatment with either DMBA or CO the skin weight per unit of area is much increased (2·07 and 2·65 times more than normal skin). The most hydrated tissue seems to be the one treated with DMBA, whereas the tissue treated with croton oil is intermediate between this and normal tissue. The greater amount of water is probably related to the increase of hyaluronic acid (HA) (see following pages): as a matter of fact an increase of HA in a given tissue determines an increase of water bound to it; histological examination of croton oil skin reveals that, although much thickened, the innermost part of the dermis is made up of closely-packed, normal collagen fibres, whereas in the case of DMBA the changes involve the whole dermis which is histochemically characterized by the presence of metachromatic material, known to be hydrophilic. The content of hexosamines per unit weight of dry material is but slightly increased in treated skin (with DMBA or with croton oil) in comparison with normal skin, in agreement with Hamer and Marchant (1957); if, however, the total amount of

TABLE II.—*Yields of Extraction of Crude MPS, and Hexosamine Content*

| | Weight of crude MPS extracted from 100 g. of dry and defatted skin (in g.) | Per cent of hexosamine content in crude MPS | Hexosamines extracted as MPS from 100 g. dry defatted skin (in mg.) | Hexosamines extracted as MPS from 1 cm.$^2$ of skin (in mg.) | Ratio between hexosamines extracted as MPS and total hexosamines of the skin |
|---|---|---|---|---|---|
| N-MPS | 1·20 | 10·35 | 124 | 0·038 | 0·38 |
| DMBA-MPS | 1·32 | 9·74 | 129 | 0·067 | 0·35 |
| CO-MPS | 1·09 | 8·5 | 93 | 0·068 | 0·26 |

N-MPS = crude MPS from normal skin.
DMBA-MPS = crude MPS from skin treated with DMBA.
CO-MPS = crude MPS from skin treated with croton oil.

hexosamines in the skin is referred to its surface, it appears to be twice as much after treatment with DMBA, and increased 2·6 times after treatment with croton oil.

Although the extraction method has been standardized, the comparison among values of the yields obtained is merely indicative : in fact the extraction yields are not constant. It may be observed that the MPS extracted are not pure (containing only 10·35 to 8·5 per cent hexosamine), and that the amount extracted, in relation to the weight of the dry defatted skin, is but slightly larger than normal in the case of DMBA skin, whereas it is less than normal in the case of croton oil skin. If referred to the surface area, the amount of MPS (estimated as hexosamines) extracted from either DMBA or croton oil skin is almost twice as much as that extracted from normal skin. The amount of hexosamine not extracted as MPS is larger in the skin treated with croton oil than in the other two cases.

In order to evaluate the glucosamine/galactosamine ratio, separations by means of column chromatography were carried out on hydrolysed MPS (Table III).

TABLE III.—*Glucosamine/Galactosamine Ratio in Crude MPS Hydrolysate*

| | |
|---|---|
| N-MPS . . | 1·01 |
| DMBA-MPS . | 3·96 |
| CO-MPS . . | 2·33 |

N-MPS = crude MPS from normal skin.
DMBA-MPS = crude MPS from skin treated with DMBA.
CO-MPS = crude MPS from skin treated with croton oil.

While in crude N-MPS the glucosamine content is but little higher than that of galactosamine, in crude DMBA-MPS the glucosamine content is almost four times as much. In the case of CO-MPS it stands between the two. (It can be remembered that HA contains glucosamine and chondroitinsulphuric acid (CSA) galactosamine).

The determination of hexosamines in the fractions derived from the elution of Hyflo blocks from electrophoresis has led to the constant observation of two peaks, one with migration towards the cathode (HA), the other one towards the anode (CSA). As a matter of fact they both migrate towards the anode, but hyaluronic acid has a lesser mobility than chondroitinsulphuric acid and seems to be shifted towards the cathode by effect of the electro-osmotic movement of the buffer.

The relative content of hexosamines in the first peak (HA) to that in the second peak (CSA) is as shown in Table IV.

TABLE IV.—*Ratio of the Amount of Hexosamines in the Slow to Fast Electrophoretic Peaks*

| | |
|---|---|
| N-MPS . . | 1·22 |
| DMBA-MPS . | 3·75 |
| CO-MPS . . | 2·80 |

These data are indicative of the interrelation between HA and CSA mucopolysaccharides. The largest amount of HA is found in MPS extracted from the skin treated with the carcinogenic agent. Identification of the first peak as HA

and of the second peak as CSA is justified by data in the literature as well as by data which will be presented later ; however, it must be considered that if MPS of heparin type were present it would not be possible to distinguish them electrophoretically from CSA because they would migrate together.

The fractionation of MPS-CPC complexes has given the following results : extraction with 2·1 N NaCl has revealed only traces of hexosamines ; the presence of measurable amounts of MPS of the heparin type can therefore be excluded. The data are thus referred to the ratio between 0·4 N fractions and 1·2 N NaCl fractions.

TABLE V.—*Ratio of the Total Amount of Hexosamine Extracted by* 0·4 N *NaCl and* 1·2 N *NaCl from MPS-CPC Complexes*

| | |
|---|---|
| N-MPS . . | 1·3 |
| DMBA-MPS . | 2·7 |
| CO-MPS . . | 2·3 |

The data reported in Table V are also indicative of the interrelation between HA and CSA, and must be examined together with those reported in Table IV.

All the MPS resulting from preparative extractions and subjected to acid hydrolysis and chromatography gave the following results : MPS from 0·4 N NaCl fraction, one peak alone : glucosamine. MPS from 1·2 N NaCl fraction, one peak of galactosamine and only traces of glucosamine.

Paper electrophoresis of MPS obtained from 1·2 N NaCl fractions (N, DMBA, CO) revealed a homogeneous rapidly migrating spot, which stained metachromatically (CSA), whereas MPS obtained from 0·4 N NaCl fractions (N, DMBA, CO) gave a large slowly migrating spot which did not stain metachromatically (HA) ; a faster migrating spot not stainable metachromatically was also observed, its mobility being but slightly inferior to that of CSA ; there were only traces of this in N and a larger amount was seen in CO and DMBA. On elution after paper electrophoresis, the acid hydrolysate of this spot did not show the presence of hexosamines.

The fraction extracted by 0·4 N NaCl is sensitive to hyaluronidase activity, whereas the 1·2 N NaCl fraction was resistant.

### CONCLUSIONS

These investigations were carried out by following several methods in combination because, although the separation and analysis of MPS has been much more widely used in the last few years, it does not yet appear advisable to rely on a single method of analysis. In fact, the comparison of the data resulting from glucosamine/galactosamine separation as well as from electrophoretic and CPC separation of crude MPS shows that there is not complete agreement : in particular, there is a considerable difference between the data obtained through CPC separation and those obtained with electrophoresis (Tables IV and V). Nevertheless they indicate that after treatment with DMBA or CO the dermis shows a very marked increase in hyaluronic acid in comparison with chondroitinsulphuric acid.

On the contrary, from a qualitative point of view, the methods employed have not shown any difference from normal, the two main types of MPS being present, that is, hyaluronic acid and chondroitinsulphuric acid. In animals treated with croton oil for the same period of time the increase in hyaluronic acid is less than in those treated with DMBA. These data are to be related to histological and histochemical observations (Prodi, 1955; Prodi and Maltoni, 1957*a*, *b*, *c*) which revealed profound changes and complete subversion of the fibrous structures in the whole dermis (with the appearance of abundant metachromatic material) after prolonged oncogenic treatment. In the course of treatment with croton oil the fibrous organisation is more superficially involved and maintains a comparative integrity in the deepest zones of the dermis in spite of remarkable alterations of the hyperplastic type and a very marked increase in the skin thickness and weight. However, it must be considered that a quantitative comparison of the results of the two treatments has limited value, since the length of the treatment and the amount and dilution of the administered substances have been chosen arbitrarily; such a comparison would be reliable if the data could be collected through the whole length of a long term experiment in the two cases.

The prevalence of hyaluronic acid over chondroitinsulphuric acid is a peculiarity of immaturity: in the pig foetus skin HA is absolutely prevailing on CSA in comparison with the adult animal skin (Loewi and Meyer, 1958); in the rat skin HA, which is very abundant in the newborn animal, is reduced to lower values in early life and changes only slightly thereafter (Prodi, unpublished data). The similarity, based on histological and histochemical observations, of skin treated with carcinogenic agents and skin in the early stages of development has been suggested by Maltoni and Prodi (1960*b*) and appears to be confirmed by the data reported in the present paper.

As far as the quantitative variations of the pool of the skin MPS are concerned, the amount per unit surface area is much increased during treatment with oncogenic and irritating substances; although when based on the dry weight of the skin, only a slight increase in total hexosamines is observed.

The various hypotheses concerning the significance of connective dermal changes (among them those on acid MPS reported here) in skin carcinogenesis have been widely discussed elsewhere (Prodi and Maltoni, 1959). The data described here show that the alterations found are not specific to the oncogenic treatment, since they can also be induced by a substance which in the rabbit is only an irritant (croton oil), even though the most active agent was shown to be DMBA. Moreover, alterations of the same type are observed in chronically sun-damaged skin (Smith *et al.*, 1961).

On the other hand it has been demonstrated from histochemical pictures that the dermis transformations are topographically in close connection with the proliferation of the overlying epithelium not only in proliferations induced by hyperplasia-promoting and irritating agents, but also in physiological conditions such as in the formation of the hair bulbs (Maltoni and Prodi, 1960*b*).

The most reasonable interpretation is, therefore, that the changes in the MPS of the dermis are part of the general picture of the alterations in the dermis which go with skin carcinogenesis and which, regardless of their being more or less specific, are nevertheless indispensable to the neoplastic process since they provide suitable surroundings for epithelial proliferation. The changes observed belong to the stage which may be called, broadly speaking, the promoting one. If we accept a two-stage theory, it is possible to suppose that the promoting action is mainly exercised on the dermis. These considerations are merely hypothetical, but they merit further investigation.

SUMMARY

In normal rabbit skin and in rabbit skin treated for one month with 9,10-dimethyl-1,2-benzanthracene or with croton oil, the total hexosamine content and the relationship between extracted acid mucopolysaccharides were analysed.

The hexosamine content was not appreciably changed in comparison with the normal, if related to tissue weight, but was considerably increased if related to skin surface.

Remarkable alterations, induced by the treatments, in the proportions of hyaluronic acid and chondroitinsulphuric acid, were noticed.

Electrophoresis on Hyflo Super-Cel and analysis with cetylpyrydinium chloride revealed a relative increase in hyaluronic acid and a decrease in chondroitinsulphuric acid.

These data were confirmed by an increase in the proportion of glucosamine to galactosamine in the hydrolysed mucopolysaccharides themselves.

Qualitative differences in mucopolysaccharides between the treated animals and controls were not revealed.

REFERENCES

Cessi, C. and Piliego, F.—(1960) *Biochem. J.*, **77**, 508.
Cramer, W. and Simpson, W. L.—(1944) *Cancer Res.*, **4**, 601.
Fels, I. G. and Greco, J.—(1961) *Ibid.*, **21**, 40.
Gardell, S.—(1953) *Acta chem. scand.*, **7**, 201.
*Idem*, Gordon, A. H. and Åqvist, S.—(1950) *Ibid.*, **4**, 907.
Hamer, D. and Marchant, J.—(1957) *Brit. J. Cancer*, **11**, 445.
van den Hooff, A.—(1962) *Oncologia*, **15**, 161.
Larsson, L. G. and Sylvén, B.—(1947) *Cancer Res.*, **7**, 676.
Loewi, G. and Meyer, K.—(1958) *Biochim. biophys. Acta.*, **27**, 453.
Ma, C. K.—(1949) *Cancer Res.*, **9**, 481.
Maltoni, C. and Prodi, G.—(1960*a*) ‘ Recent contributions to cancer in Italy ’, Milano (Casa Editrice Ambrosiana).—(1960*b*) *Tumori*, **46**, 28.
Meyer, K.—(1956) *Biochim. biophys. Acta*, **21**, 506.
Orr, J. W.—(1938) *J. Path. Bact.*, **46**, 495.
Prodi, G.—(1955) *Nature, Lond.*, **175**, 1130.
*Idem* and Maltoni, C.—(1957*a*) *J. Path. Bact.*, **73**, 355.—(1957*b*) *Tumori*, **43**, 430.—(1957*c*) *Ibid.*, **43**, 466.—(1959) ‘ Stato attuale delle conoscenze sulle alterazioni connettivali nell’oncogenesi e sul loro significato ’. Milano (Casa Editrice Ambrosiana).
Schiller, S., Mathews, M. B., Jefferson, H., Ludowieg, J. and Dorfman, A.—(1954) *J. biol. Chem.*, **211**, 717.
*Idem*, Slover, G. A. and Dorfman, A.—(1961) *Ibid.*, **236**, 983.
Scott, J. E.—(1960) *Meth. biochem. Analysis*, **8**, 145.
Smith, J. G., Davidson, E. A., Tindall, J. P. and Sams, W. M.—(1961) *Proc. Soc. exp. Biol. N.Y.*, **108**, 533.
Sylvén, B.—(1954) *Acta Un. int. Cancr.*, **10**, 169.
*Idem* and Larsson, L. G.—(1948) *Cancer Res.*, **8**, 449.